# Mild dopa-responsive dystonia in heterozygous *tyrosine hydroxylase* mutation carrier: Evidence of symptomatic enzyme deficiency?

**DOI:** 10.1016/j.parkreldis.2020.01.017

**Published:** 2020-02

**Authors:** Julien F. Bally, David P. Breen, Susen Schaake, Joanne Trinh, Aleksandar Rakovic, Christine Klein, Anthony E. Lang

**Affiliations:** Department of Neurology, University of Geneva and University Hospitals of Geneva, Geneva, Switzerland; Edmond J. Safra Program in Parkinson's Disease and Morton and Gloria Shulman Movement Disorders Clinic, Toronto Western Hospital, Toronto, Canada; Edmond J. Safra Program in Parkinson's Disease and Morton and Gloria Shulman Movement Disorders Clinic, Toronto Western Hospital, Toronto, Canada; Centre for Clinical Brain Sciences, University of Edinburgh, Edinburgh, UK; Anne Rowling Regenerative Neurology Clinic, University of Edinburgh, Edinburgh, UK; Usher Institute of Population Health Sciences and Informatics, University of Edinburgh, Edinburgh, UK; Institute of Neurogenetics, University of Lübeck, Lübeck, Germany; Edmond J. Safra Program in Parkinson's Disease and Morton and Gloria Shulman Movement Disorders Clinic, Toronto Western Hospital, Toronto, Canada

**Keywords:** Dystonia, Dopa-responsive, Tyrosine hydroxylase, Heterozygote, Autosomal recessive

## Abstract

We present a case of mild, adult-onset dopa-responsive dystonia (DRD) with a heterozygous mutation in the tyrosine hydroxylase (*TH*) gene. We propose that this genetic state may have led to partial enzyme deficiency. Future studies should attempt to identify and characterize the phenotype of other patients with single *TH* variants.

## 1 Manuscript

Dopa-responsive dystonia (DRD) refers to a group of hereditary dystonias that improve with levodopa. Heterozygous mutations affecting the guanosine triphosphate cyclohydrolase (*GCH1*) gene cause the most common and mildest form of DRD, with dystonic features typically manifesting in childhood. Autosomal recessive *GCH1* cases tend to be more severe [1]. Biallelic mutations in the sepiapterin reductase (*SPR*), 6-pyruvoyl tetrahydropterin (*PTP*) synthase and tyrosine hydroxylase (*TH*) genes typically lead to more severe phenotypes and present earlier in life. In *TH* deficiency, isolated dystonia is uncommon; patients have parkinsonism and/or a complex disorder combining dystonia, other movement disorders (tremor, myoclonus, oculogyric crisis) plus additional features [1]. Patients with *TH* deficiency have previously been classified as either type A which manifests in the first few years of life (range 2 months–5 years) and is characterized by a progressive hypokinetic-rigid syndrome with dystonia, or type B which manifests sooner after birth (range 0–3 months) with a more complex encephalopathy (including the varied movements disorders described above plus intellectual impairment and autonomic dysfunction) [2]. Here, we report a patient with mild, adult-onset DRD with two heterozygous variants on the same allele of the *TH* gene. Written informed consent for publication was obtained from the patient.

A 65-year-old man developed left foot dystonia without diurnal fluctuations aged 38 years. There was no history of other complaints such as oculogyric crises or autonomic dysfunction. Two years later, levodopa-carbidopa 100/25mg 1 tablet once daily resulted in a dramatic and sustained response, allowing him to continue working as a post-office courier until his planned retirement. He had no problems with motor dexterity. He retained mildly abnormal posturing of his fingers, mild stiffness of his legs (particularly on the left side), neither of which improved with higher levodopa dosage. On three occasions, levodopa withdrawal resulted in gradual re-emergence of foot dystonia over months. He never developed dyskinesias or motor fluctuations in response to levodopa.

He had no children. His deceased father, examined in his 80s, had some jerky hand movements but no neurological diagnosis was made. His deceased mother had isolated excessive eye blinking throughout most of her adult life.

When last examined, he had clawing of the toes, left-sided jerks of individual fingers, semi-rhythmic movements of the hands when arms were outstretched (consistent with dystonic tremor), mildly increased tone in both upper limbs, but no bradykinesia. Gait was normal.

Fluorodopa PET imaging performed aged 41 was normal. The patient declined CSF neurotransmitter testing. No mutations in *GCH1* or *SPR* genes were found. The *GCHI*, *TH*, and *SPR* genes were analyzed by PCR and sequencing of both DNA strands of the entire coding region and the highly conserved exon-intron splice junctions. Multiplex ligation-dependent probe amplification (MLPA) analyses were performed for *GCHI* and *TH*. *TH* gene analysis showed a pathogenic variant (c.296del [p.Leu99Argfs*15]) and a variant of uncertain significance (c.1169C  >  T [p.Ser390Leu]), which were confirmed to be present on the same allele using a comprehensive third-generation sequencing approach (see Supplemental Methods).

To our knowledge, only biallelic *TH*-DRD cases have been reported to date [3]. A 2010 study described 36 *TH*-DRD cases and reviewed all previously published cases: the oldest age of onset was 5 years [2]. Since then, others have reported onset at older ages (7, 12 and 18 years) [[3], [4], [5]]. If the present *TH* mutation is the cause of our patient's DRD, he has by far the oldest age of onset and is the only one with a mild phenotype of isolated dystonia resembling *GCH1*-DRD.

As in some other recessive disorders, we hypothesize that heterozygosity in *TH*-DRD could lead to a benign form of a typically severe disease, possibly due to a mild reduction in TH activity. Supporting this hypothesis, a small but significant reduction in striatal levodopa was observed in the post-mortem brain tissue of a heterozygous knock-in mouse model replicating a human *TH* mutation, as opposed to the profound reduction seen in the homozygous mouse model, demonstrating that the effect may be dependent on gene dosage [6]. Further studies of adult-onset DRD cases without *GCH1* mutations may identify similar cases and provide further insights into the genetic spectrum of this condition.

## 2 Authors' roles

Julien F. Bally: Conception, Organization, Execution, Writing of the first draft. David P. Breen: Organization, Execution, Review and Critique. Susen Schaake: Organization, Execution, Review and Critique. Joanne Trinh: Execution, Review and Critique. Aleksandar Rakovic: Execution, Review and Critique. Christine Klein: Organization, Execution, Review and Critique. Anthony E. Lang: Conception, Organization, Review and Critique.

## Full financial disclosures of all authors for the past year

JFB: 2019 JNLF congress fee paid by Abbvie and partial 2019 PRD congress fee paid by Zambon.

DPB: Consultancies: Closed Loop Medicine. Honoraria: Abbvie, Bial and GE Healthcare; Grants: Wellcome Trust Clinical Research Career Development Fellowship and RS MacDonald Neurological Seedcorn Fund; Employment: University of Edinburgh and NHS Lothian.

SS: No financial disclosures.

JT: No financial disclosures.

AR: No financial disclosures.

CK: serves as a medical consultant to Centogene for genetic testing in the field of movement disorders and dementia, excluding Parkinson's disease. Stock Ownership in medically-related fields: None. Advisory Boards: Advisory Boards. Partnerships: None. Honoraria: Annals of Neurology (Associate Editor). Grants: DFG, BMBF, EU, MJFF, CCXDP. Intellectual Property Rights: None. Expert Testimony: None. Employment: University of Luebeck. Contracts: None. Royalties: Oxford University Press. Other: None.

AEL: Consultancies: Abbvie, Acorda, AFFiRis, Biogen, Bristol Myers Squibb, Intracellular, Janssen, Jazz, Lilly, Lundbeck, Merck, Ono, Paladin, Roche, Seelos, Syneos, Sun Pharma, Theravance, and Corticobasal Degeneration Solutions; Advisory Boards: Jazz Pharma, PhotoPharmics, Sunovion; Honoraria:Sun Pharma, AbbVie and Sunovion: Grants: Brain Canada, Canadian Institutes of Health Research, Corticobasal Degeneration Solutions, Edmond J Safra Philanthropic Foundation, Michael J. Fox Foundation, the Ontario Brain Institute, Parkinson Foundation, Parkinson Canada, and W. Garfield Weston Foundation; Royalties: Elsevier, Saunders, Wiley-Blackwell, Johns Hopkins Press, and Cambridge University Press.

## Declaration of competing interest

The manuscript has not been previously published and is not under review at any other journal. No other related work is under submission elsewhere. All authors of the paper have participated to the study, revised the manuscript and approved the final version of the manuscript. There is no ghost writer. There is no financial or any other type of conflict of interest related to the manuscript.

## References

[1] Wijemanne S., Jankovic J. (2015). Dopa-responsive dystonia--clinical and genetic heterogeneity. Nat. Rev. Neurol..

[2] Willemsen M.A., Verbeek M.M., Kamsteeg E.-J. (2010). Tyrosine hydroxylase deficiency: a treatable disorder of brain catecholamine biosynthesis. Brain: J. Neurol..

[3] Furukawa Y., Kish S. Tyrosine hydroxylase deficiency.; 1993. http://www.ncbi.nlm.nih.gov/pubmed/20301610.

[4] Yeung W.-L., Wong V.C.N., Chan K.-Y. (2011). Expanding phenotype and clinical analysis of tyrosine hydroxylase deficiency. J. Child Neurol..

[5] Katus L.E., Frucht S.J. (2017). An unusual presentation of tyrosine hydroxylase deficiency. J. clin. Mov. Disord..

[6] Rose S.J., Yu X.Y., Heinzer A.K. (2015). A new knock-in mouse model of l-DOPA-responsive dystonia. Brain: J. Neurol..

