# Supplementary material for: Mild dopa-responsive dystonia in heterozygous tyrosine hydroxylase mutation carrier: Evidence of symptomatic enzyme deficiency?
Source: Parkinsonism Relat Disord. 2020 Feb;71:44–5. doi: 10.1016/j.parkreldis.2020.01.017 (PMC7109519; doi:10.1016/j.parkreldis.2020.01.017)
Supplement: Multimedia component 1 [file mmc1.docx]

# Supplemental methods

To investigate whether these TH gene variants were present on the same or different alleles, the whole-length open reading frame of the TH gene was amplified using cDNA transcribed from the patient and a healthy control. The PCR product was digested with the NheI restriction enzyme and cloned into a pcDNA 3.1 plasmid (ThermoFisher), followed by transformation in OneShot E.coli (ThermoFisher). 20 individual colonies were picked, transferred into LB medium and incubated overnight. The plasmid DNA from each colony was extracted and the whole product was sequenced on an ABI3130XL (Applied Biosystems). Two TH isoforms (transcript ID ENST00000381178.1 and ENST00000381175.1) were found in the plasmid DNA. However, both clones carried only the wildtype allele for the two detected variants. We postulate that the two mutations are in cis and undergo non-sense mediated decay due to the frameshift variant (c.296del [p.Leu99Argfs*15]).

To provide further confirmation, we additionally amplified a 4.16 kb sequence in the *TH* gene, which covered nine coding exons and the introns between them (chr11: 2,187,042-2,197,623). Primer sequences were 5’-TCATCCCCTGCCTCTGTGT-3’ and 5’-TCACCCGTGACCAGGATAC-3’. Q5 High Fidelity 2x Master Mix (New England Biolabs) with appropriate optimized PCR conditions (98°C 30, 35x (94°C 10s, 57°C 10s, 72°C 2 min), 72°C 1 min) were used for amplification. The PCR product was completely loaded on a 1.5% agarose gel. After 45 min running time at 120V, the right sized band was cut out and extracted using the QIAquick gel extraction kit (QIAGEN). The Oxford Nanopore MinION was used to perform long-read sequencing with the 1D ligation kit (SQK-LSK109) on the R9.4.1 flow cell. MinKNOW version 2.0 was used for data acquisition. Guppy version 5.02.05 was used for basecalling and generating fast5 and fastq files. Fast5 files were converted to fastq with NanoOK^1^ or Poretools^2^. We used Nanoplot for quality control and visualization. After filtering the data by mean q90 with filtlong, we aligned reads to the human genome (hg19) with Minimap2 and performed variant calling using GraphMap or NGMLR. Samtools (version 1.3.1) was used to merge, sort and index the bam files. The data were viewed on IGV. The longest read length achieved for the amplicon of interest was 4.16kb, and the reads produced showed that the p.Leu99Argfs*15 and p.Ser390Leu were in cis (i.e. on the same allele), thus confirming the cloning results.

# References

1. Leggett RM, Heavens D, Caccamo M, Clark MD, Davey RP. NanoOK: multi-reference alignment analysis of nanopore sequencing data, quality and error profiles. *Bioinformatics*. 2016;32(1):142-144. doi:10.1093/bioinformatics/btv540

2. Loman NJ, Quinlan AR. Poretools: a toolkit for analyzing nanopore sequence data. *Bioinformatics*. 2014;30(23):3399-3401. doi:10.1093/bioinformatics/btu555
